# Supplementary material for: A Novel Chaperone-Based Cancer Vaccination Enhances Immunotherapeutic Responsiveness Through T Cell Amplification and Tumor Immune Remodeling
Source: Vaccines (Basel). 2025 Oct 25;13(11):1096. doi: 10.3390/vaccines13111096 (PMC12656611; doi:10.3390/vaccines13111096)
Supplement: Supplementary file 1 [file vaccines-13-01096-s001.zip › vaccines-3917108-supplementary.pptx]

## Slide 1
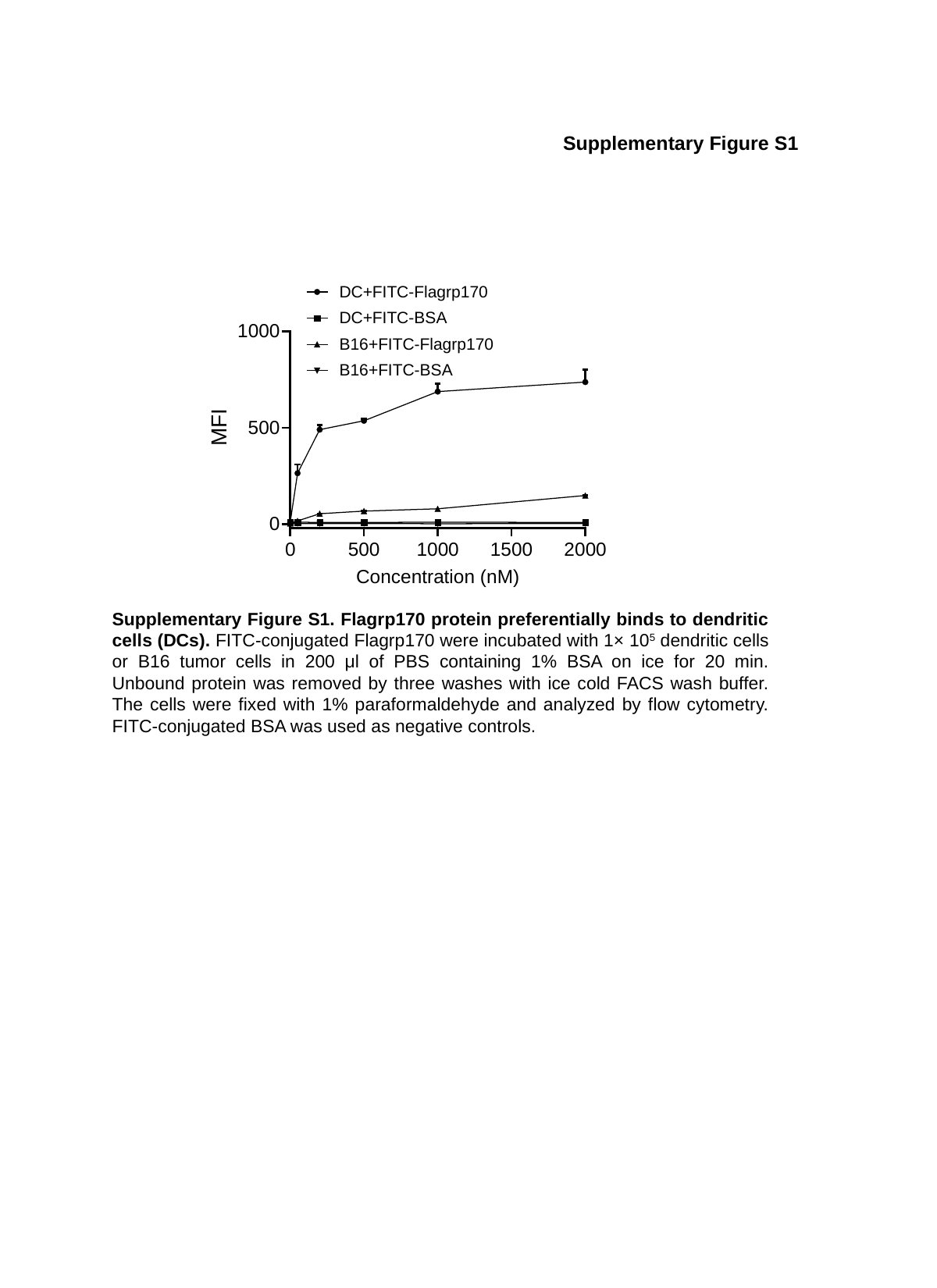

Supplementary Figure S1
Supplementary Figure S1. Flagrp170 protein preferentially binds to dendritic cells (DCs). FITC-conjugated Flagrp170 were incubated with 1× 10^5^ dendritic cells or B16 tumor cells in 200 μl of PBS containing 1% BSA on ice for 20 min. Unbound protein was removed by three washes with ice cold FACS wash buffer. The cells were fixed with 1% paraformaldehyde and analyzed by flow cytometry. FITC-conjugated BSA was used as negative controls.

## Slide 2
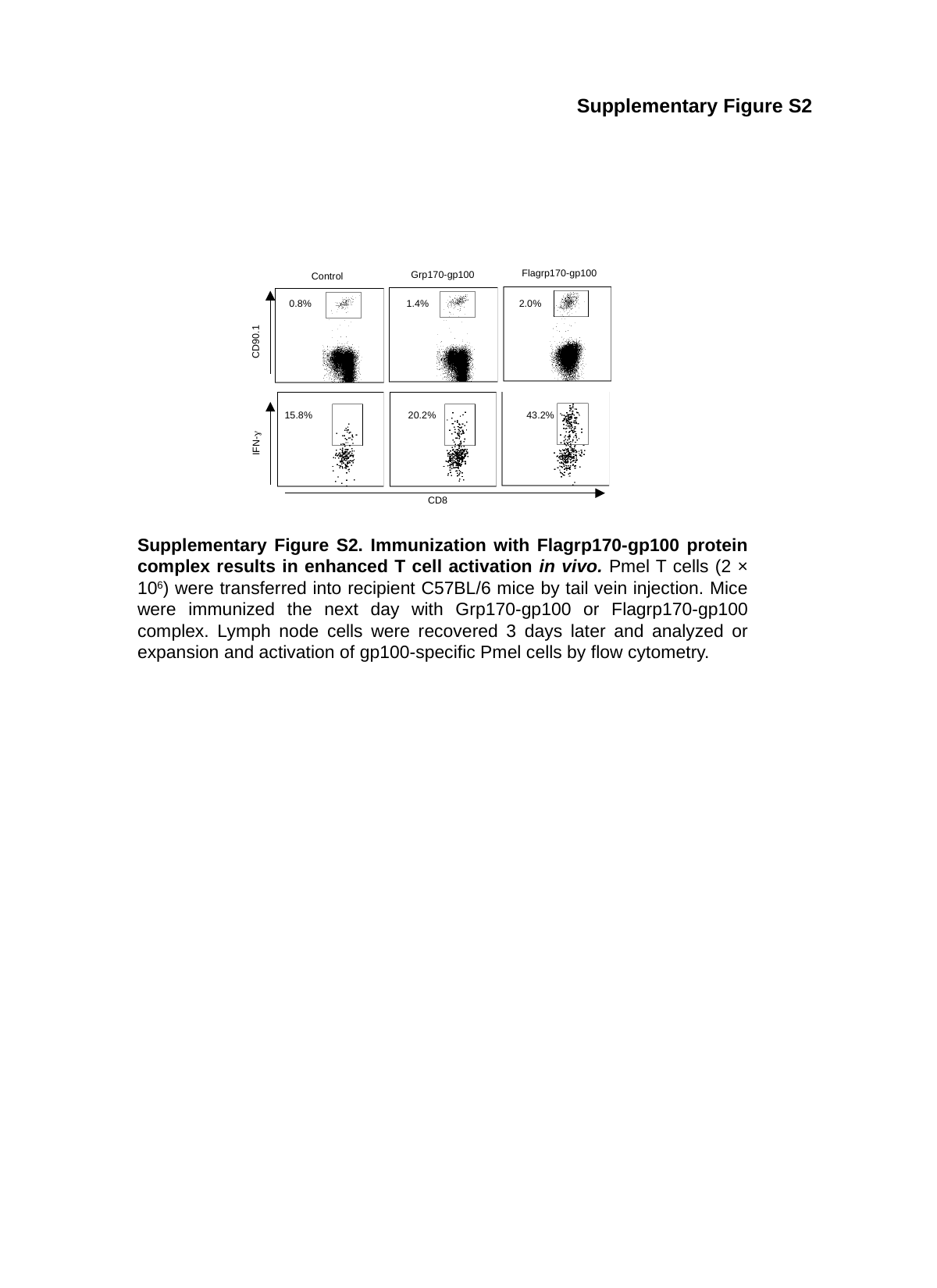

Supplementary Figure S2
Flagrp170-gp100
Grp170-gp100
Control
0.8%
2.0%
1.4%
CD90.1
43.2%
15.8%
20.2%
IFN-γ
CD8
Supplementary Figure S2. Immunization with Flagrp170-gp100 protein complex results in enhanced T cell activation in vivo. Pmel T cells (2 × 10^6^) were transferred into recipient C57BL/6 mice by tail vein injection. Mice were immunized the next day with Grp170-gp100 or Flagrp170-gp100 complex. Lymph node cells were recovered 3 days later and analyzed or expansion and activation of gp100-specific Pmel cells by flow cytometry.

## Slide 3
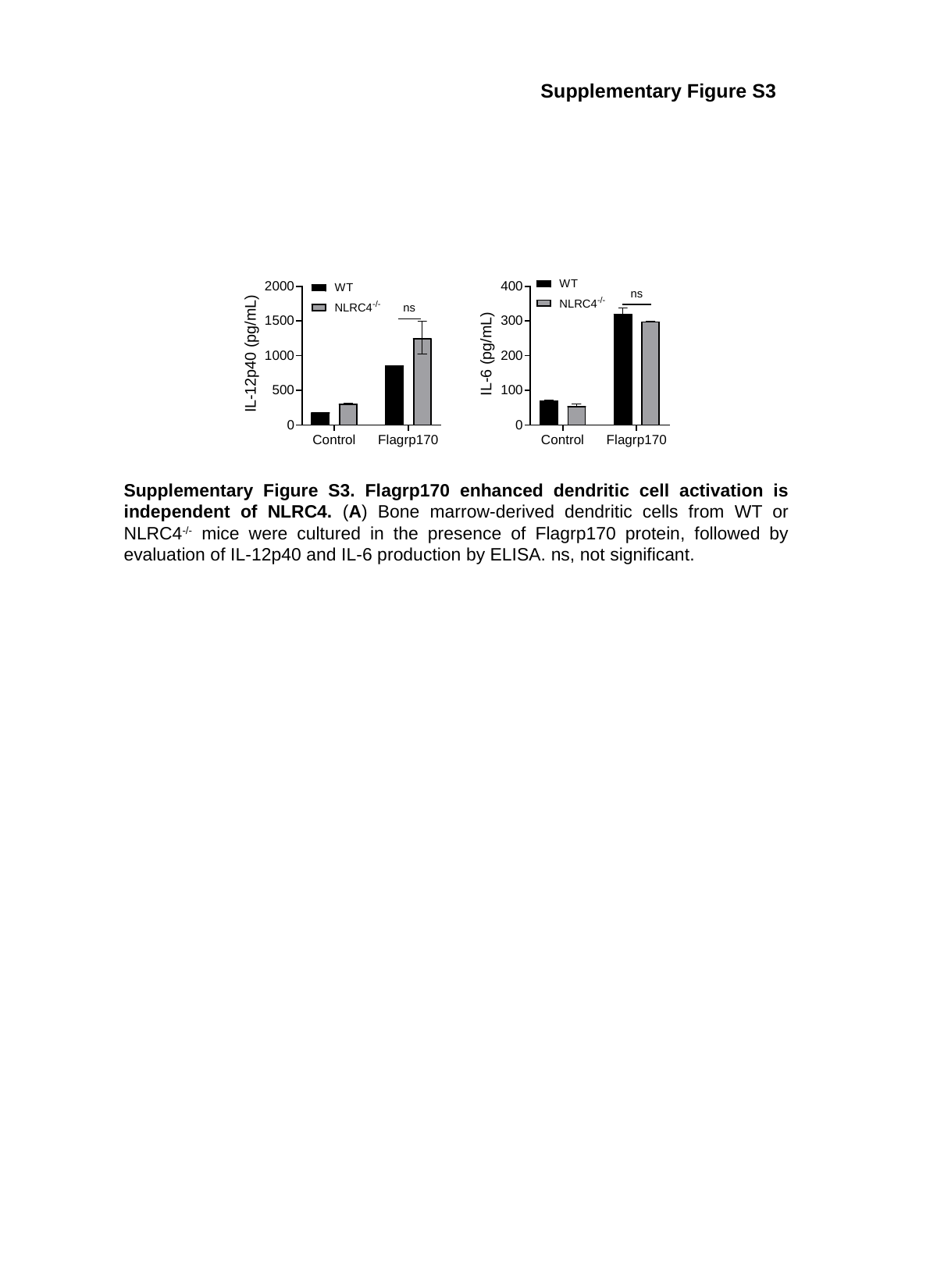

Supplementary Figure S3
Supplementary Figure S3. Flagrp170 enhanced dendritic cell activation is independent of NLRC4. (A) Bone marrow-derived dendritic cells from WT or NLRC4-/- mice were cultured in the presence of Flagrp170 protein, followed by evaluation of IL-12p40 and IL-6 production by ELISA. ns, not significant.

## Slide 4
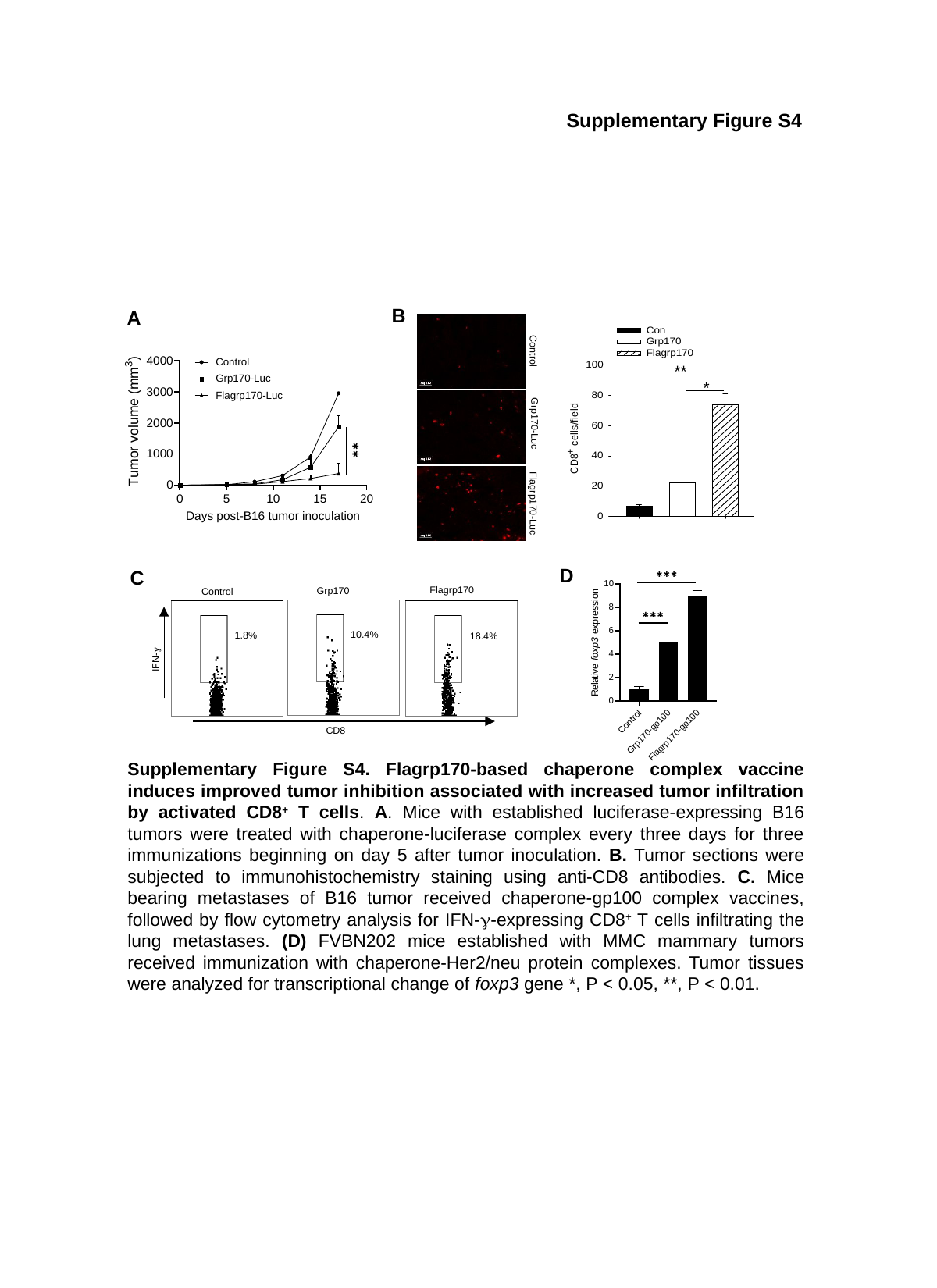

Supplementary Figure S4
B
A
**
*
Control
Grp170-Luc
Flagrp170-Luc
D
C
Flagrp170
Grp170
Control
10.4%
1.8%
18.4%
IFN-γ
CD8
Supplementary Figure S4. Flagrp170-based chaperone complex vaccine induces improved tumor inhibition associated with increased tumor infiltration by activated CD8+ T cells. A. Mice with established luciferase-expressing B16 tumors were treated with chaperone-luciferase complex every three days for three immunizations beginning on day 5 after tumor inoculation. B. Tumor sections were subjected to immunohistochemistry staining using anti-CD8 antibodies. C. Mice bearing metastases of B16 tumor received chaperone-gp100 complex vaccines, followed by flow cytometry analysis for IFN-γ-expressing CD8+ T cells infiltrating the lung metastases. (D) FVBN202 mice established with MMC mammary tumors received immunization with chaperone-Her2/neu protein complexes. Tumor tissues were analyzed for transcriptional change of foxp3 gene *, P < 0.05, **, P < 0.01.

## Slide 5
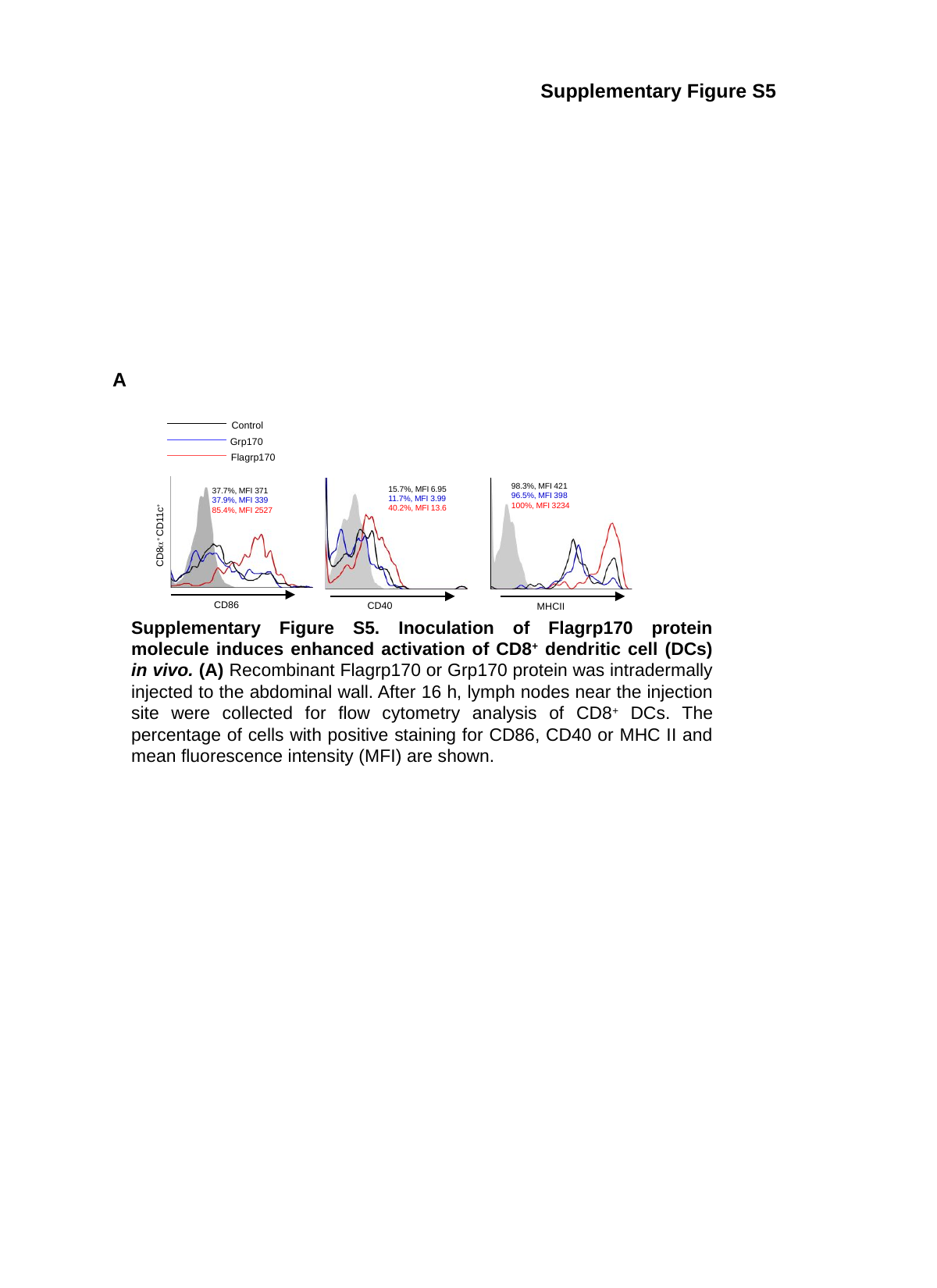

Supplementary Figure S5
A
Control
Grp170
Flagrp170
98.3%, MFI 421
96.5%, MFI 398
100%, MFI 3234
15.7%, MFI 6.95
11.7%, MFI 3.99
40.2%, MFI 13.6
37.7%, MFI 371
37.9%, MFI 339
85.4%, MFI 2527
CD8α+ CD11c+
CD86
CD40
MHCII
Supplementary Figure S5. Inoculation of Flagrp170 protein molecule induces enhanced activation of CD8+ dendritic cell (DCs) in vivo. (A) Recombinant Flagrp170 or Grp170 protein was intradermally injected to the abdominal wall. After 16 h, lymph nodes near the injection site were collected for flow cytometry analysis of CD8+ DCs. The percentage of cells with positive staining for CD86, CD40 or MHC II and mean fluorescence intensity (MFI) are shown.
