# Supplementary material for: A Novel Chaperone-Based Cancer Vaccination Enhances Immunotherapeutic Responsiveness Through T Cell Amplification and Tumor Immune Remodeling
Source: Vaccines (Basel). 2025 Oct 25;13(11):1096. doi: 10.3390/vaccines13111096 (PMC12656611; doi:10.3390/vaccines13111096)
Supplement: Supplementary file 1 [file vaccines-13-01096-s001.zip › Supplementary File S1.pptx]

## Slide 1
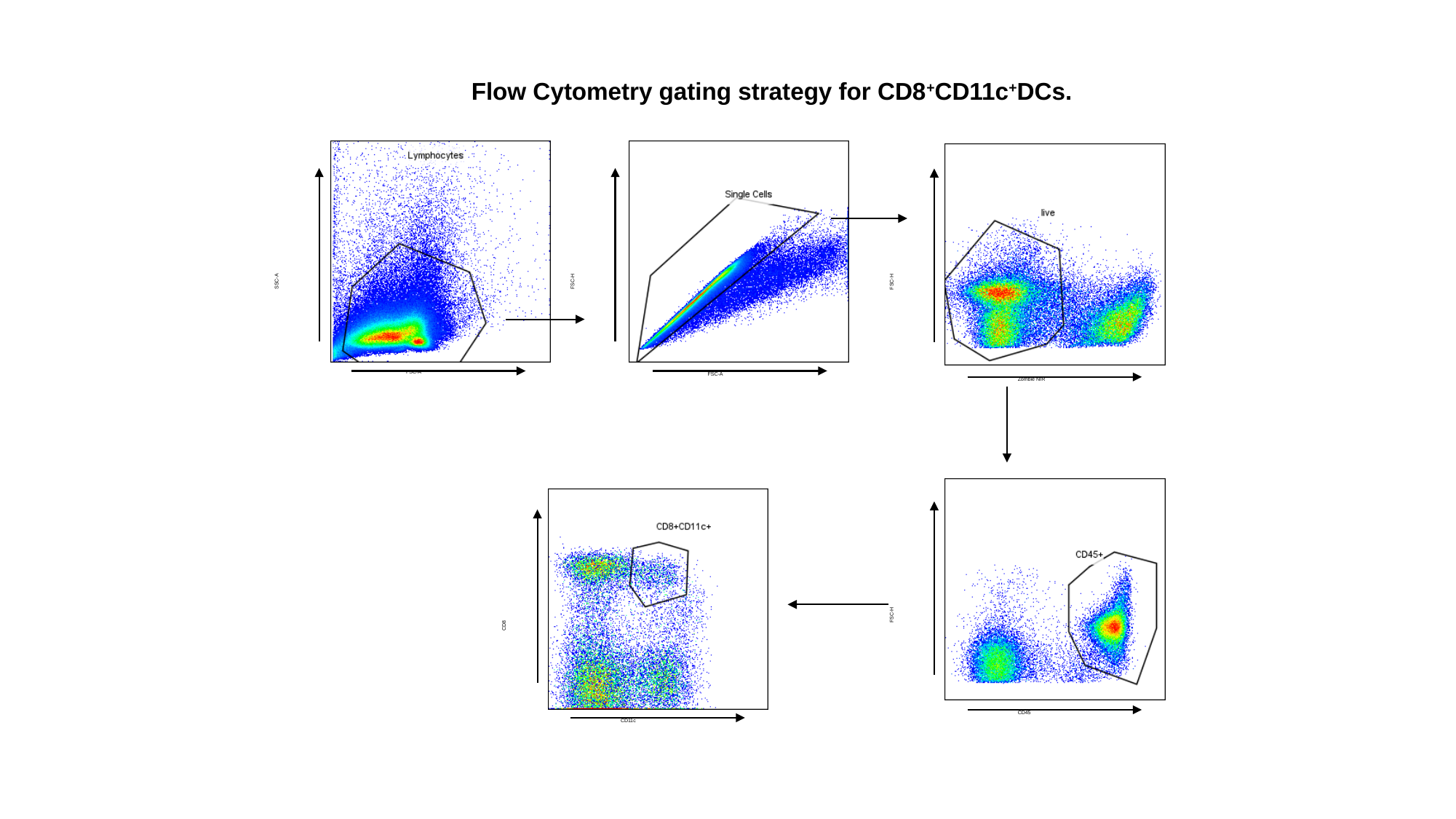

Flow Cytometry gating strategy for CD8+CD11c+DCs.
SSC-A
FSC-H
FSC-H
FSC-A
FSC-A
Zombie NIR
FSC-H
CD8
CD45
CD11c
