# Supplementary material for: A Novel Chaperone-Based Cancer Vaccination Enhances Immunotherapeutic Responsiveness Through T Cell Amplification and Tumor Immune Remodeling
Source: Vaccines (Basel). 2025 Oct 25;13(11):1096. doi: 10.3390/vaccines13111096 (PMC12656611; doi:10.3390/vaccines13111096)
Supplement: Supplementary file 1 [file vaccines-13-01096-s001.zip › Supplementary File S2.pptx]

## Slide 1
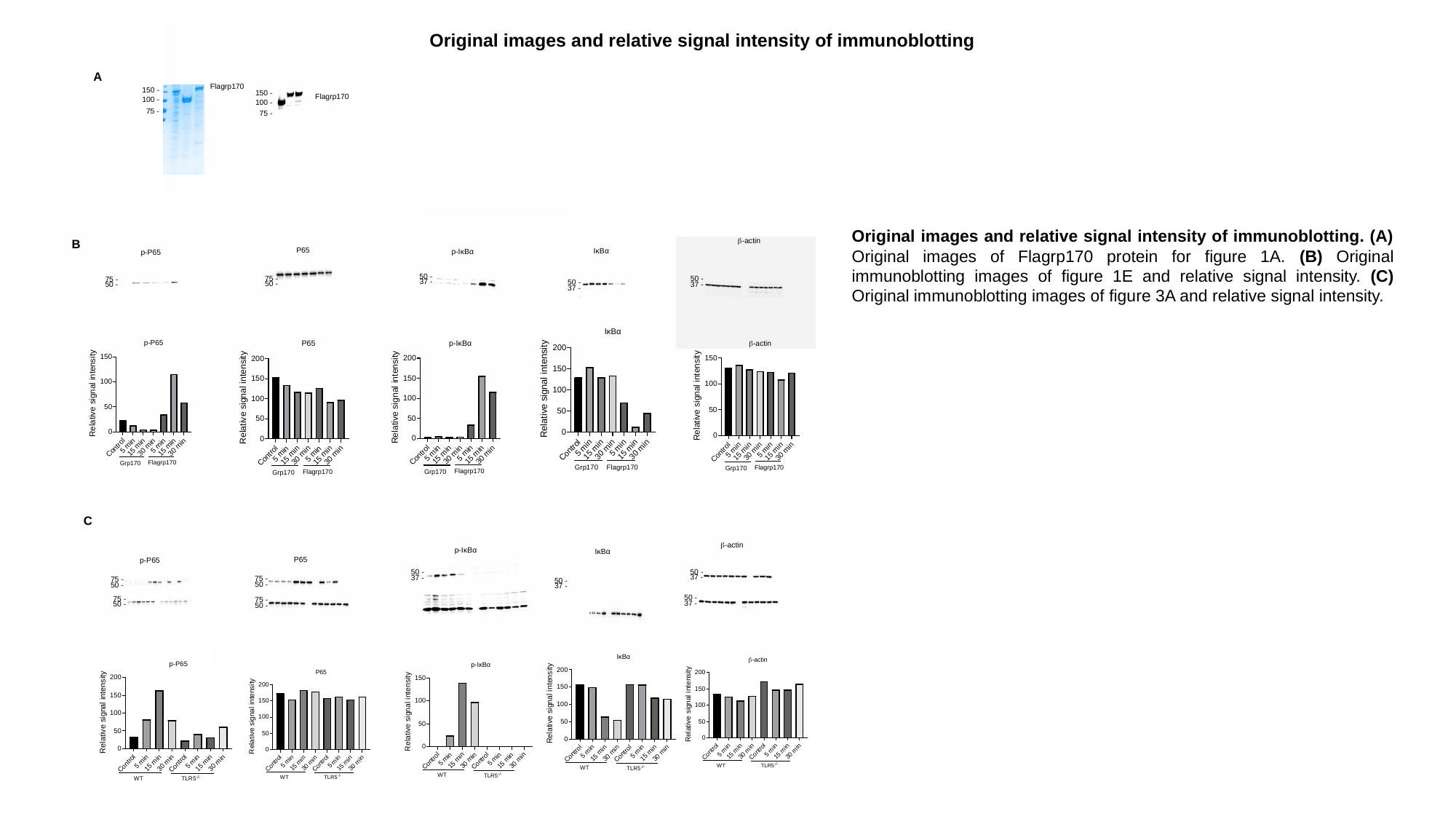

Flagrp170
150 -
100 -
75 -
150 -
100 -
75 -
Flagrp170
Original images and relative signal intensity of immunoblotting
A
Original images and relative signal intensity of immunoblotting. (A) Original images of Flagrp170 protein for figure 1A. (B) Original immunoblotting images of figure 1E and relative signal intensity. (C) Original immunoblotting images of figure 3A and relative signal intensity.
β-actin
B
P65
IκBα
p-IκBα
p-P65
50 -
37 -
75 -
50 -
50 -
37 -
75 -
50 -
50 -
37 -
C
β-actin
p-IκBα
IκBα
P65
p-P65
50 -
37 -
50 -
37 -
75 -
50 -
75 -
50 -
50 -
37 -
50 -
37 -
75 -
50 -
75 -
50 -
